# Supplementary material for: Single-cell mapping of lipid metabolites using an infrared probe in human-derived model systems
Source: Nat Commun. 2024 Jan 8;15:350. doi: 10.1038/s41467-023-44675-0 (PMC10774263; doi:10.1038/s41467-023-44675-0)
Supplement: Supplementary file 1 — Supplementary Information [file 41467_2023_44675_MOESM1_ESM.pdf]

Supplementary Information

Single-cell mapping of lipid metabolites using an infrared probe in human-derived model systems

Yeran Bai[1,2,*], Carolina M. Camargo[1], Stella Glasauer[1], Raymond Gifford[1], Xinran Tian[1], Andrew P. Longhini[1], Kenneth S. Kosik[1,*]

[1]Neuroscience Research Institute, Department of Molecular, Cellular, and Developmental Biology, University of California, Santa Barbara, CA 93106, USA

[2]Photothermal Spectroscopy Corp., Santa Barbara, CA 93101, USA

[*]Corresponding authors: yrbai@ucsb.edu (Y.B.), kosik@lifesci.ucsb.edu (K.S.K.)

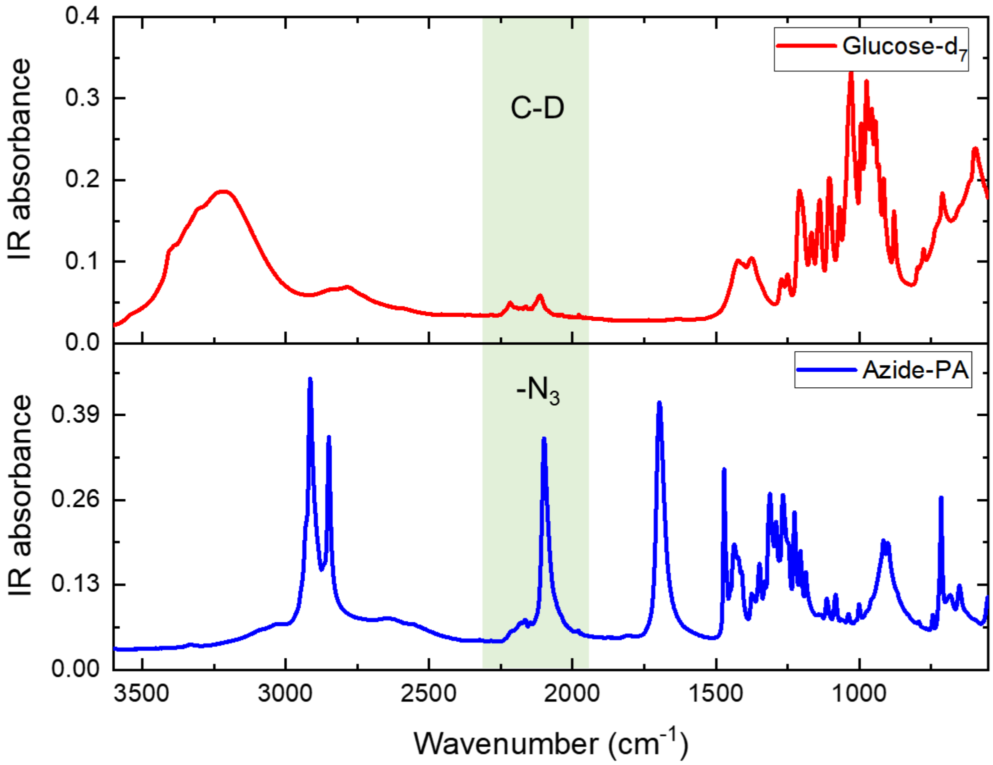

Supplementary Figure 1. Fourier transform infrared (FTIR) spectra of glucose-d$_7$ and azide-PA powder. The spectral region of interest is highlighted with a green box in the cell-silent region. It is clear the -N_3 stretching mode is significantly stronger compared to the C-D stretching mode. The spectra were taken on an attenuated total reflectance-FTIR spectrometer with the 128 averages of both background and signal acquisition.

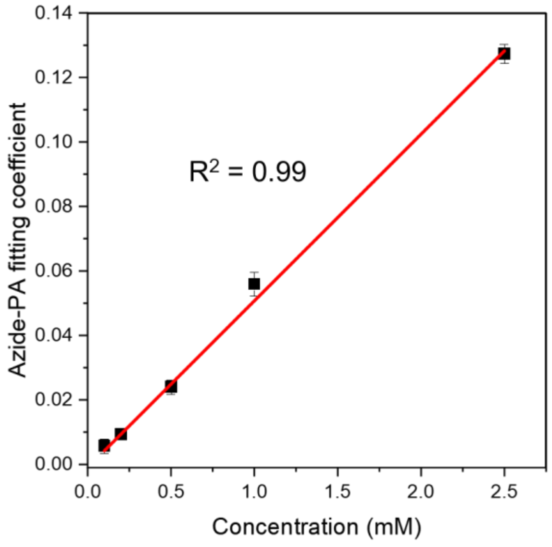

Supplementary Figure 2. Determination of azide-PA detection limit. Azide-PA powder was dissolved in dimethyl sulfoxide (DMSO) at serial dilutions of 2.5 mM, 1 mM, 500 µM, 200 µM, and 100 µM. The solution was sandwiched between two substrates (top: 0.17 mm-thick glass coverslip, bottom: 1 mm-thick CaF_2 plate). Counter propagation (visible focused from top and IR focused from bottom) and transmission detection were used. Spectra were acquired in the range of 2040 to 2300 cm^{-1}. Pure DMSO and azide-PA spectra were acquired to unmix the contribution from azide-PA and DMSO. Linear unmixing was performed to extract the coefficient of azide-PA. At least three locations were acquired for each concentration, and the mean and standard deviation of the azide-PA fitting coefficient are plotted in the curve. At 100 µM, the signal-to-noise ratio (calculated by dividing the mean by standard deviation) is 2.5. The mean value was linear fitted and plotted in red curve.

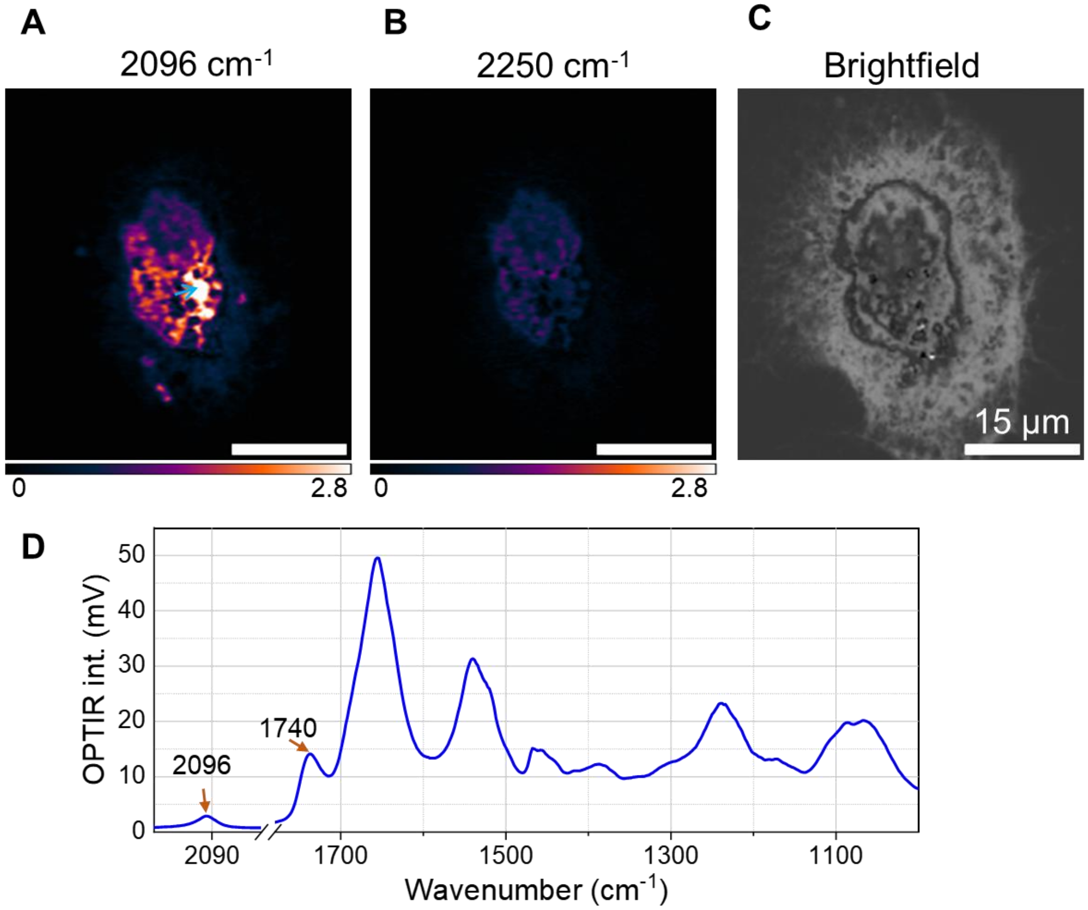

Supplementary Figure 3. OPTIR imaging with lower azide-PA incubation concentration. Neuroglioma H4 cells were incubated with azide-PA at 20 µM (final concentration) for 24 h before imaging. (**A-C**) Representative OPTIR images at indicated channels. Compared with the newly-synthesized lipid image (2096 cm^{-1}), the off-resonance image (2250 cm^{-1}) showed much diminished contrast. (**D**) The pinpointed (location displayed with blue arrow in (A)) spectrum showed distinctive total lipid (1740 cm^{-1}) and newly-synthesized peak (2096 cm^{-1}), confirming the successful incorporation of azide tags into intracellular lipids.

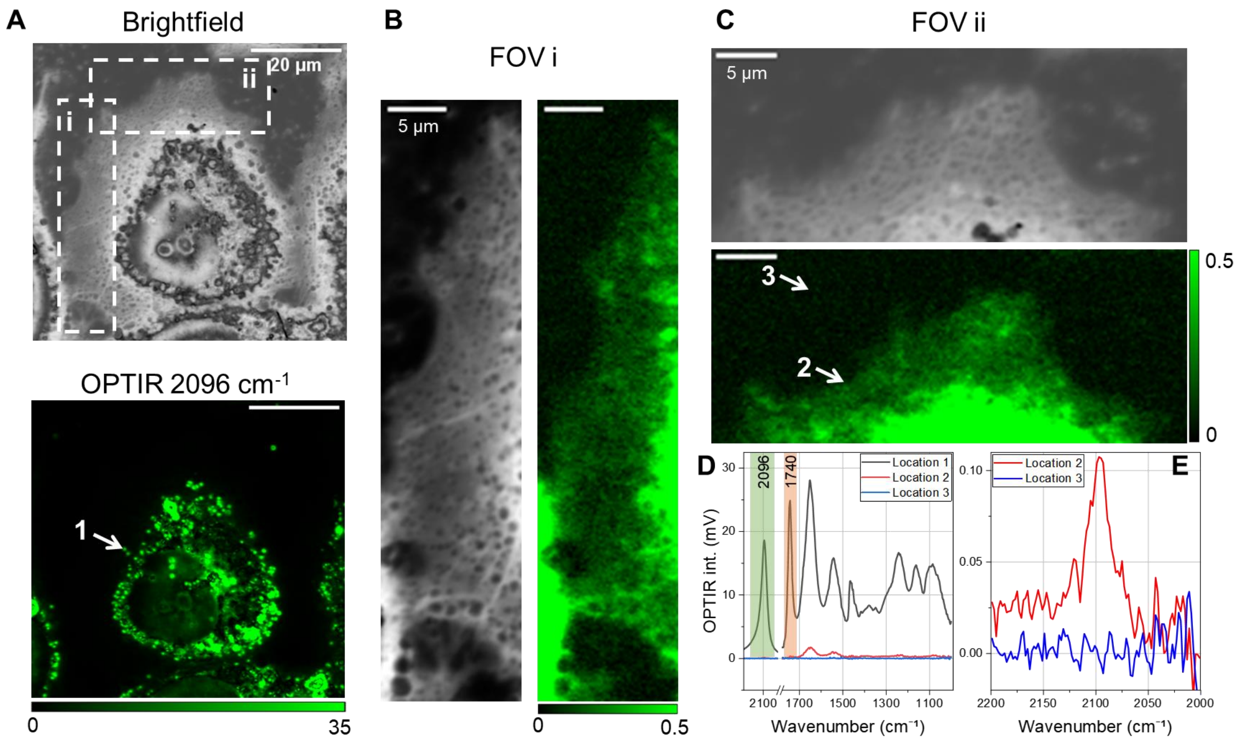

Supplementary Figure 4. Imaging of azide incorporation into plasma membranes via OPTIR. (A) Representative brightfield and OPTIR imaging at newly-synthesized lipid channel (2096 cm^{-1}) of human neuroglioma H4 cells. The contrast scale for OPTIR 2096 cm^{-1} is 0 to 35 mV. The cells were incubated in azide-PA containing media for 6.5 hr before fixed for imaging. (**B-C**) Zoom-in view of the regions indicated in panel (A) with white dashed squares. The contrast scale for the OPTIR 2096 cm^{-1} channel is 0 to 0.5 mV. The cell boundaries are visible at 2096 cm^{-1}, indicating the azide-tagged lipids have successfully incorporated into plasma membranes. (**D**) Pinpoint spectra at locations indicated in (A) and (C). Location 1 (black curve) is a lipid droplet, location 2 (red curve) is on the cell boundary, and location 3 (blue curve) is a no-cell region. (**E**) Zoom-in spectra of the 2000 to 2200 cm^{-1} region for cell boundary and no-cell region. A clear peak centered around 2096 cm^{-1} is visible on the cell boundary location but not in the no-cell region, further confirming the presence of azide-tagged lipids at cell membranes.

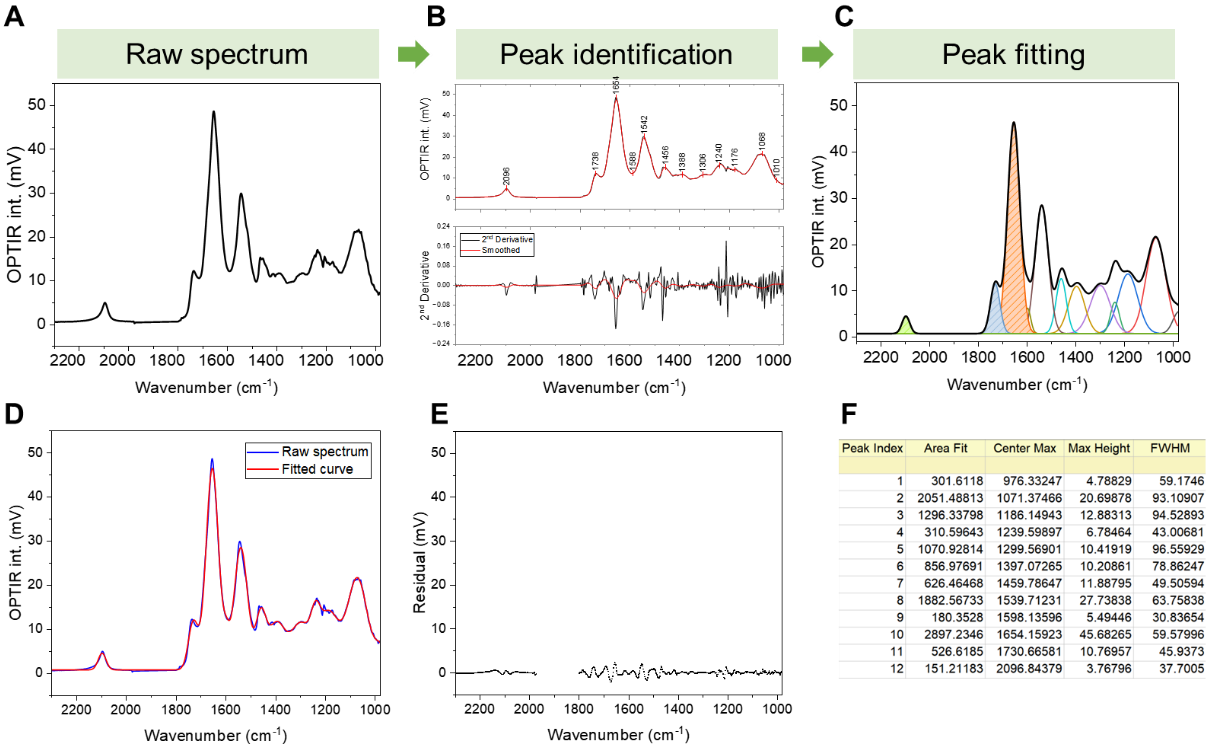

Peak Index	Area Fit	Center Max	Max Height	FWHM
1	301.6118	976.33247	4.78829	59.1746
2	2051.48813	1071.37466	20.69878	93.10907
3	1296.33798	1186.14943	12.88313	94.52893
4	310.59643	1239.59897	6.78464	43.00681
5	1070.92814	1299.56901	10.41919	96.55929
6	856.97691	1397.07265	10.20861	78.86247
7	626.46468	1459.78647	11.88795	49.50594
8	1882.56733	1539.71231	27.73838	63.75838
9	180.3528	1598.13596	5.49446	30.83654
10	2897.2346	1654.15923	45.68265	59.57996
11	526.6185	1730.66581	10.76957	45.9373
12	151.21183	2096.84379	3.76796	37.7005

Supplementary Figure 5. Spectral fitting procedure for quantifications. An example raw spectrum from azide-PA incubated cell is shown in **(A)**. Spectral peaks were found using smoothed second derivative of the raw spectrum. In the top panel of **(B)**, the smoothed raw spectrum is shown with a red curve, which was obtained through the Savizky-Golay smoothing method using a window size of 19. Meanwhile, the black curve in the bottom panel at (B) represents the second derivative, and the red curve shows the smoothed second derivative, which was achieved through a smoothing method using a Savizky-Golay 2nd polynomial function with a window length of 31 pts. Peaks identified with this method were indicated on the curve in top panel of (B). Gauss function was used for curve peak fitting. **(C)** Spectral fitting result. Each fitted curve based on the located peak is shown in color with the accumulative curve shown in black. The peaks used for quantifications in this study were protein peak (~1650 cm^{-1}, orange), total lipid peak (~ 1740 cm^{-1}, blue), and newly-synthesized lipid peak (~2096 cm^{-1}, green). **(D-E)** Comparison of the raw and fitted curve, and the fitting residual. **(F)** List of the fitting results for all the peaks. The peak fitting analysis was performed with OriginPro 2022b software with the Peak Analyzer function.

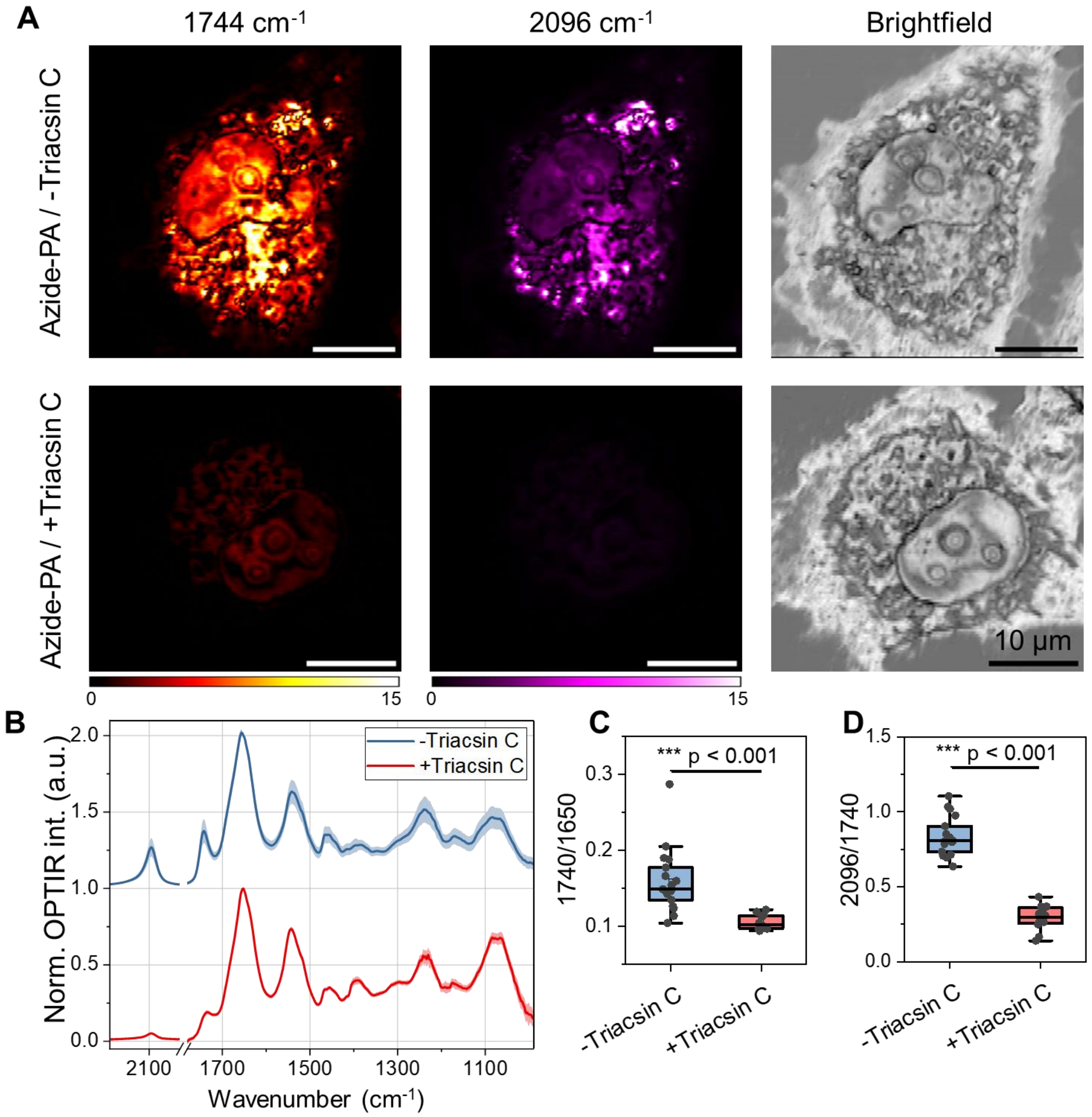

Supplementary Figure 6. Triacsin C treatment significantly reduces the normalized total lipid and newly-synthesized to total lipids ratio. The control cells were incubated with 100 µM (final concentration) azide-PA containing media for 24 hr. For the Triacsin C treatment group, 1 µM of Triacsin C was added together with the azide-PA and incubated for 24 hr. (**A**) Representative OPTIR images at indicated wavenumbers and corresponding brightfield images. (**B**) Spectra from two groups. Mean (solid curve) and standard deviation (shaded area) are derived from -Triacsin C (n = 12) and +Triacsin C (n = 17) groups. Spectra were offset for clarity. (**C-D**) Statistical quantification of normalized total lipids (1740/1650) and newly-synthesized to total lipids ratio (2096/1740) for -Triacsin C (n = 12) and +Triacsin C (n = 17) groups. Statistical test: two-sided two-sample t-test (C-D). The box plot displays the median (central line), 25th, and 75th percentiles of the data, with the outliers identified using a coefficient of 1.5 times the interquartile range (C-D).

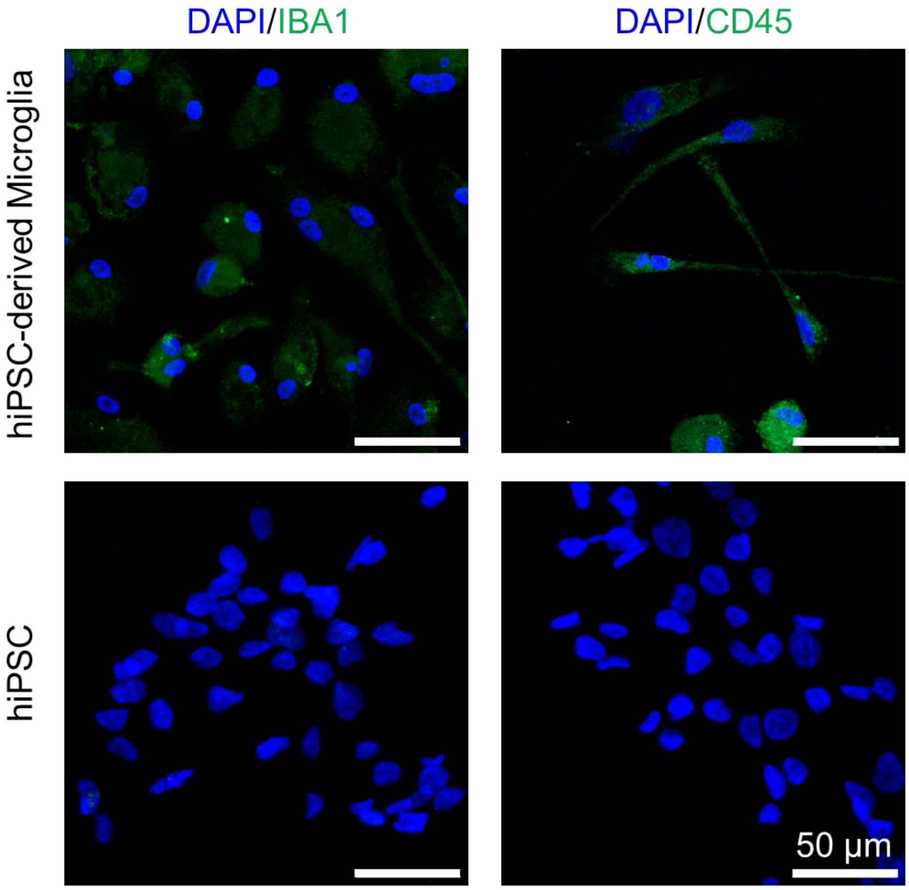

Supplementary Figure 7. Representative confocal fluorescence imaging of hiPSC-derived microglia and hiPSCs immunostained for canonical microglia markers IBA1 and CD45.

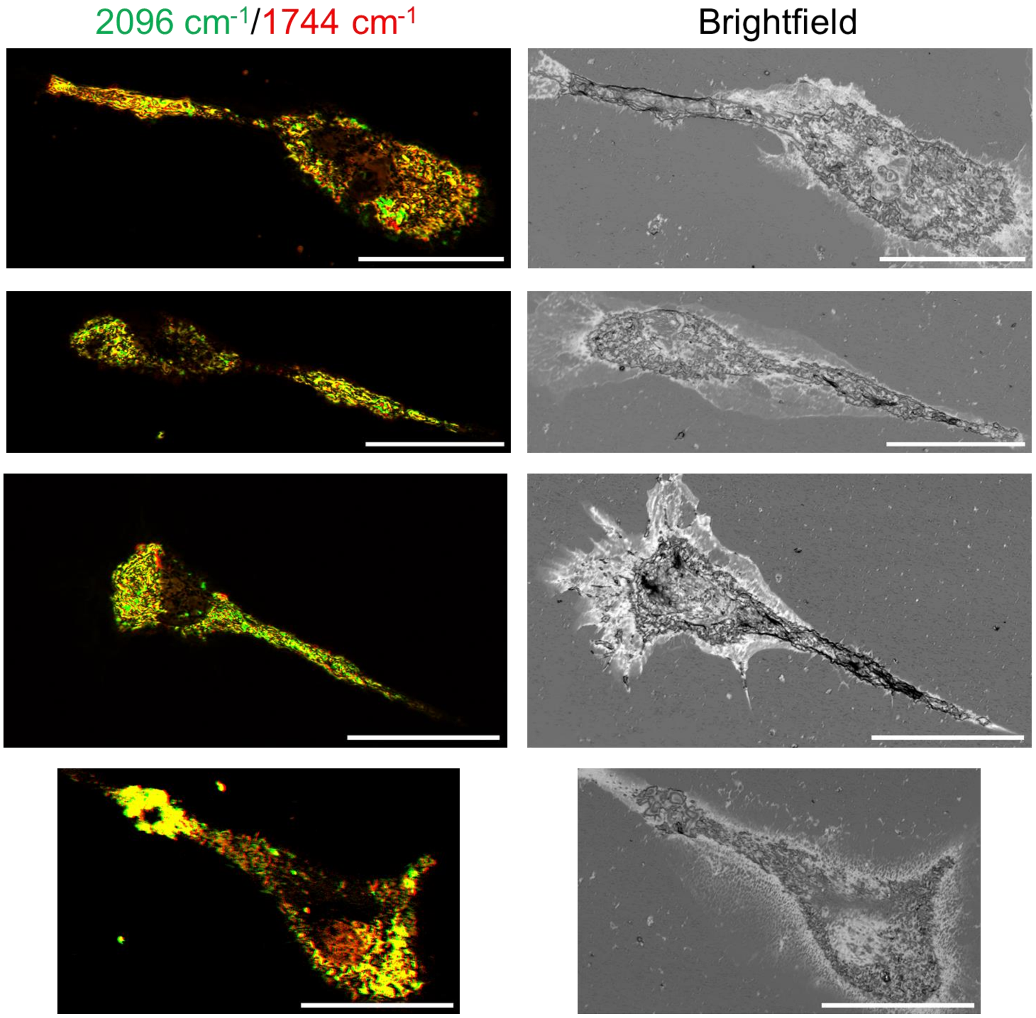

Supplementary Figure 8. Newly-synthesized lipids (2096 cm^{-1}) largely overlap with the total lipids (1744 cm^{-1}). Representative field of view (FOV) of merged OPTIR images and brightfield images. Scale bars, 50 μm.

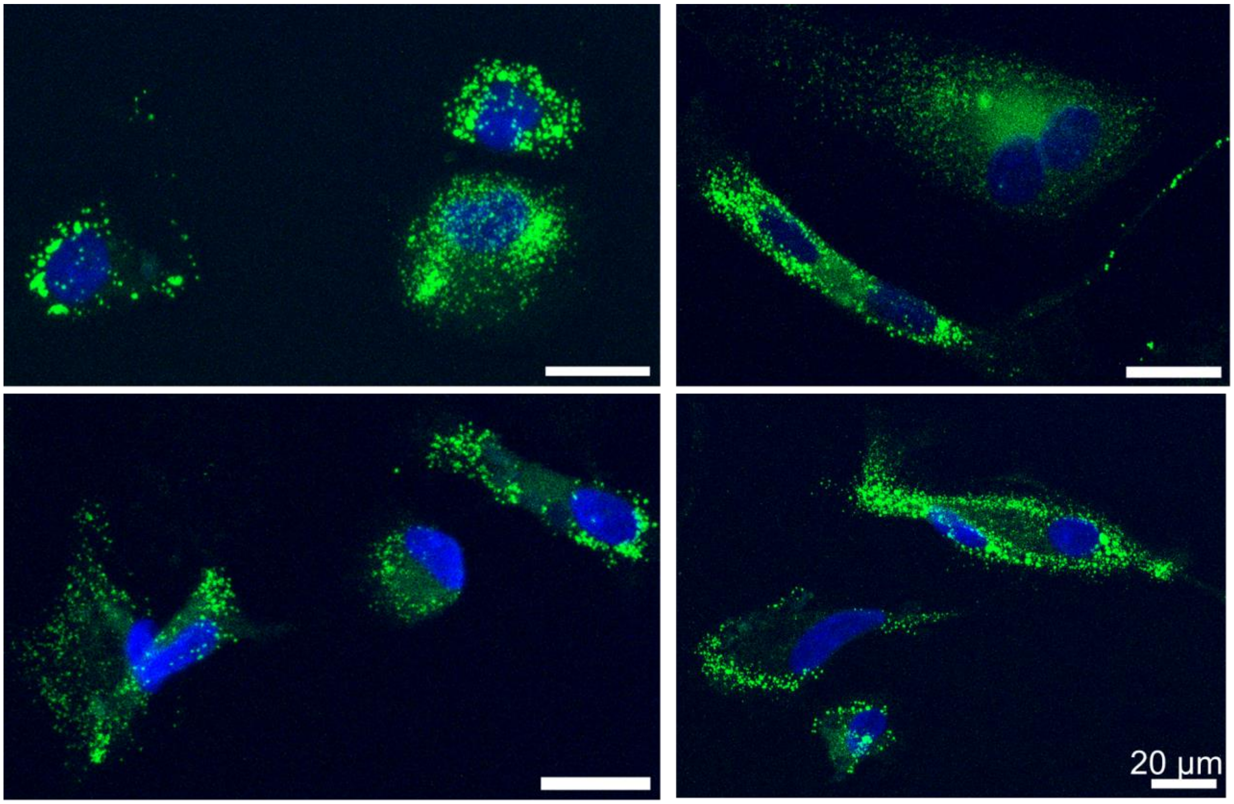

Supplementary Figure 9. Representative confocal fluorescence images of hiPSC-derived microglia. The cells were not treated with additional fatty acids. Blue for DAPI and green for BODIPY 493/503.

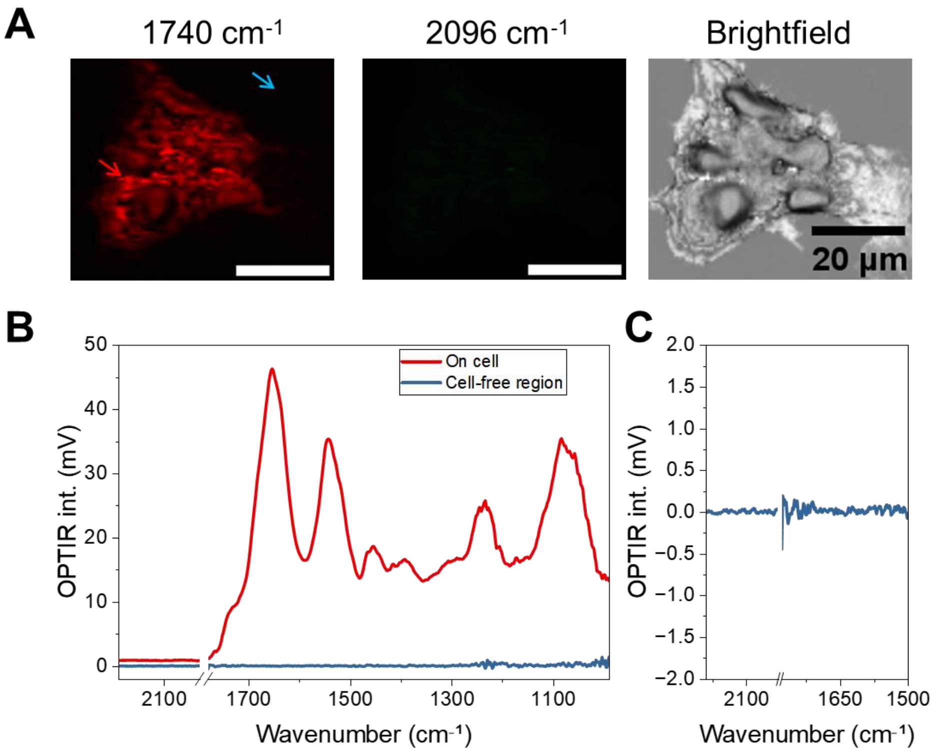

Supplementary Figure 10. Coating with Matrigel to support stem cell growth does not interfere with lipid metabolic imaging. (A) Representative images of no azide-PA treated stem cells at the total lipid peak (1740 cm^{-1}), the newly-synthesized lipid peak (2096 cm^{-1}), and the brightfield image. **(B)** Raw spectra were acquired on the cell (red) and a cell-free region (blue). The location where the spectra were acquired is indicated with red and blue arrows in (A). **(C)** Zoom-in view of the cell-free region spectrum shows no observable peaks.

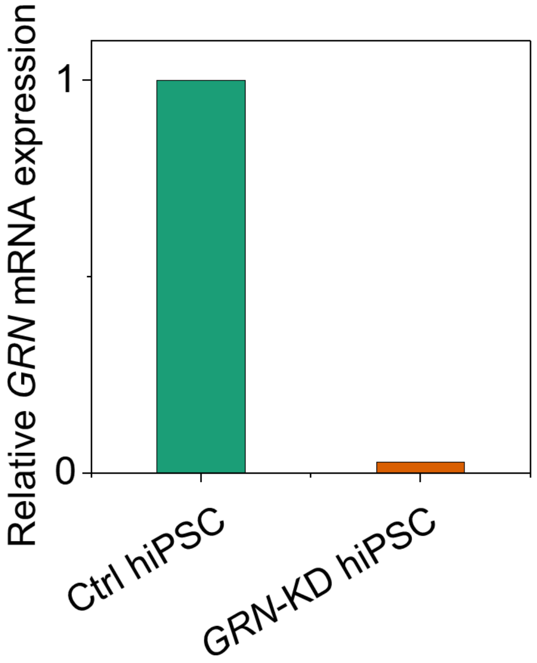

Supplementary Figure 11. qPCR characterization of *GRN* expression levels in Ctrl and *GRN*-KD hiPSCs confirmed successful knockdown of *GRN*.

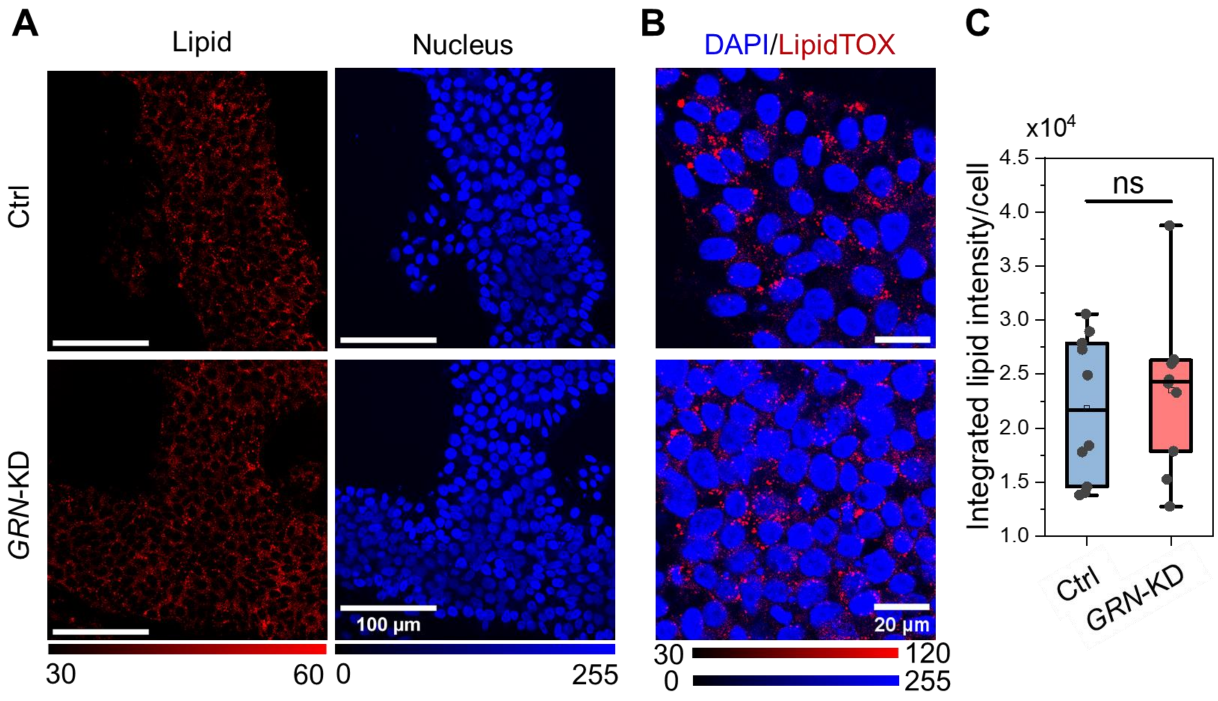

Supplementary Figure 12. Confocal fluorescence imaging of control and *GRN*-KD hiPSC showed non-significant different total lipids contents. (A) Representative confocal fluorescence images of control and *GRN*-KD hiPSCs. The cells were treated with 100 µM azide-PA for 24 hours, fixed, stained with LipidTOX Red (Invitrogen H34477), and mounted with Prolong Diamond Antifade Mountant with DAPI (Invitrogen P36962). (**B**) Zoom-in views to better show lipid distribution inside cells. **(C)** Quantification of total lipids. Thresholding masks of lipid contrasts were first created to remove the background signal, and the threshold (pixel value ≥ 30) was consistent for different FOVs as well as different cell lines. The raw lipid images were then multiplied by the corresponding masks and the total intensity within an FOV was integrated. To eliminate the influence of different cell counts on the quantification, we normalized the integrated lipid intensity with the nuclei counts (obtained from DAPI images) in the same FOV. Each dot in (B) represents a FOV with a dimension of 290 µm by 290 µm, and the cell counts across different FOVs and cell lines range from 190 to 378. Ten FOVs were used for each cell line. The box plot displays the median (central line), 25th, and 75th percentiles of the data. The statistical test is two-sided two-sample t-test.

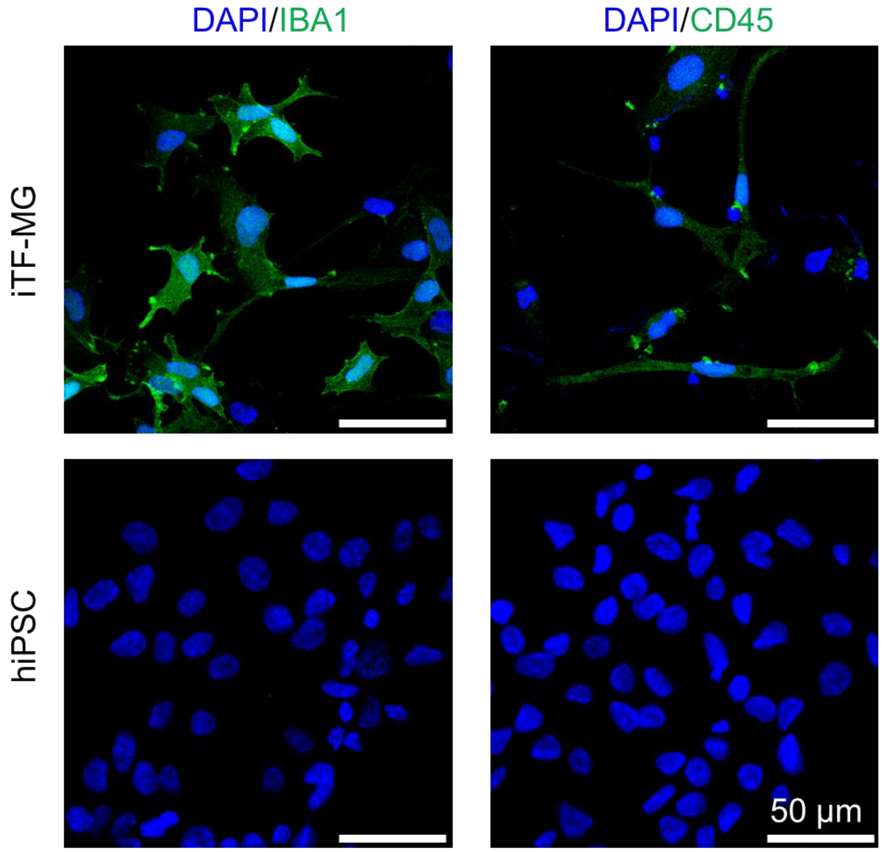

Supplementary Figure 13. Representative confocal fluorescence images of induced-transcription factor microglia-like cells (iTF-MG) and hiPSCs immunostained for canonical microglia markers IBA1 and CD45.

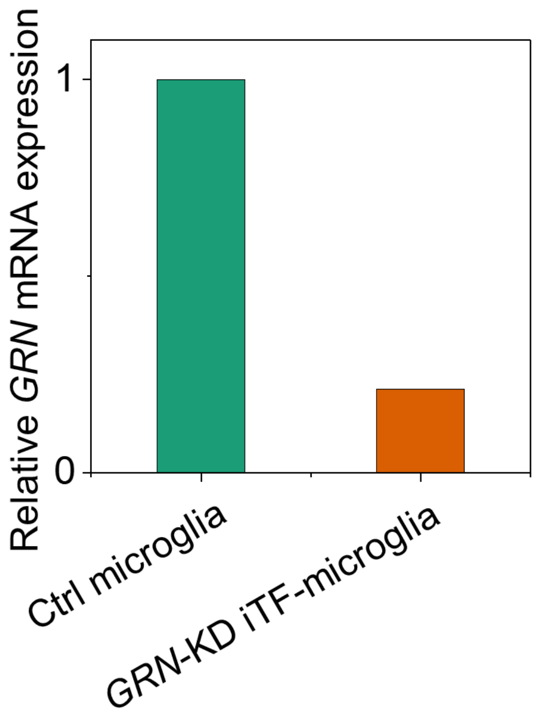

Supplementary Figure 14. qPCR characterization of *GRN* expression levels in Ctrl and *GRN*-KD induced-transcription factor (iTF)-microglia confirmed successful knockdown of *GRN*.

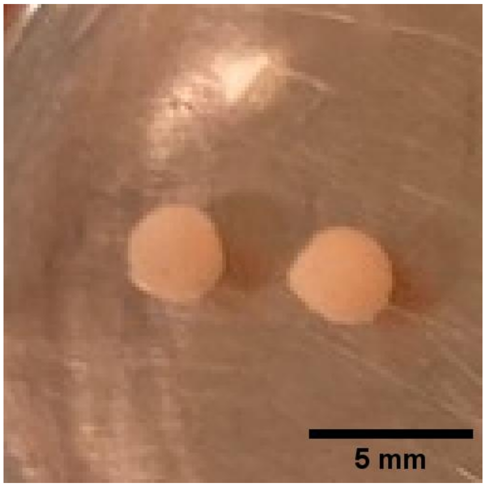

Supplementary Figure 15. Brightfield images of brain organoids. The organoids were differentiated, cultured, and maintained with the same protocol described in the main manuscript. The image was taken at 6-month cultivation.

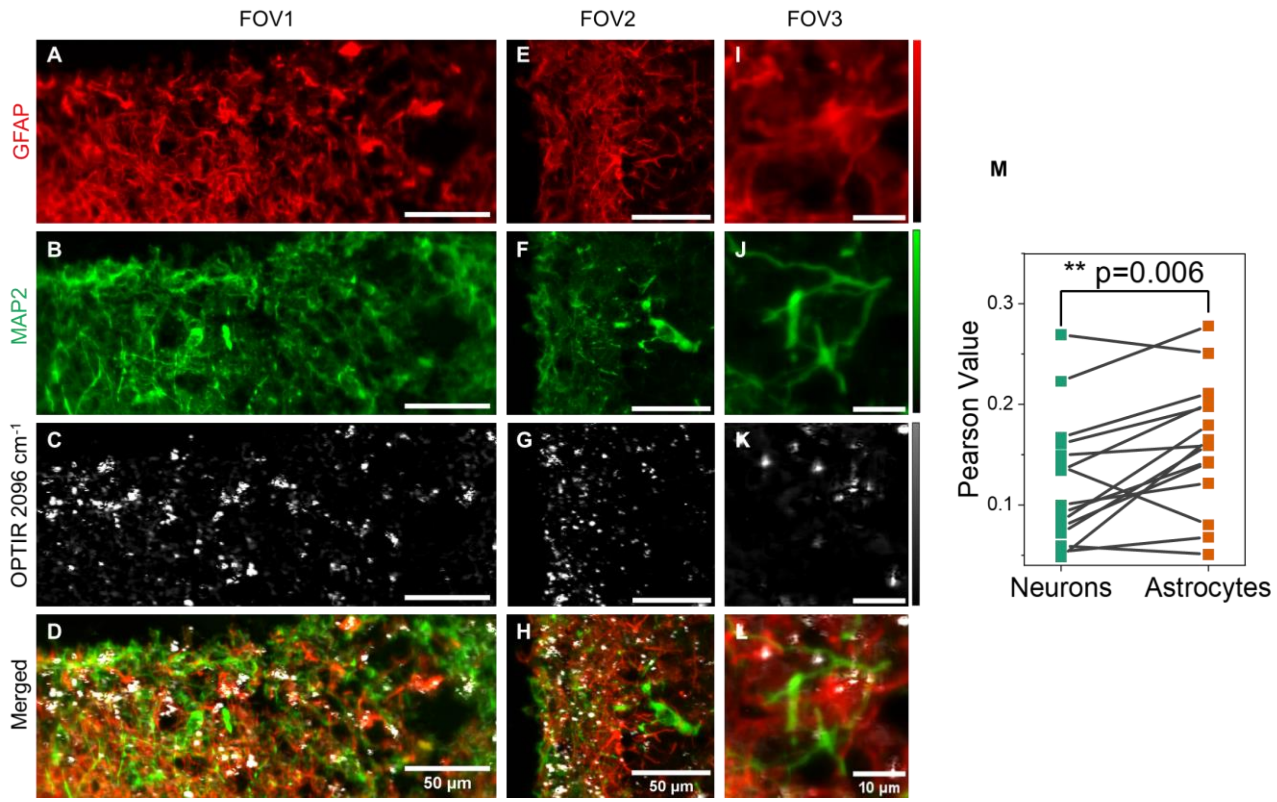

Supplementary Figure 16. Astrocytes exhibit higher newly-synthesized lipid levels compared to that of neurons. (**A-L**) Representative FOVs for MAP2 (neuron marker), GFAP (astrocyte marker), OPTIR azide, and merged images. (**M**) Pearson correlation value showed significantly higher colocalization of newly-synthesized lipids with astrocytes than neurons. Each pair represents a FOV with dimensions in the range of 120 to 290 μm (n = 15). Statistical test: two-sided paired sample t-test.
