# Supplementary material for: Single-cell mapping of lipid metabolites using an infrared probe in human-derived model systems
Source: Nat Commun. 2024 Jan 8;15:350. doi: 10.1038/s41467-023-44675-0 (PMC10774263; doi:10.1038/s41467-023-44675-0)
Supplement: Supplementary file 3 — Reporting Summary [file 41467_2023_44675_MOESM3_ESM.pdf]

nature portfolio

Corresponding author(s): Yeran Bai, Kenneth Kosik

Last updated by author(s): Dec 1, 2023

Reporting Summary

Nature Portfolio wishes to improve the reproducibility of the work that we publish. This form provides structure for consistency and transparency in reporting. For further information on Nature Portfolio policies, see our Editorial Policies and the Editorial Policy Checklist.

Statistics

For all statistical analyses, confirm that the following items are present in the figure legend, table legend, main text, or Methods section.

n/a	Confirmed	
☐	☒	The exact sample size (*n*) for each experimental group/condition, given as a discrete number and unit of measurement
☐	☒	A statement on whether measurements were taken from distinct samples or whether the same sample was measured repeatedly
☐	☒	The statistical test(s) used AND whether they are one- or two-sided<br>*Only common tests should be described solely by name; describe more complex techniques in the Methods section.*
☒	☐	A description of all covariates tested
☒	☐	A description of any assumptions or corrections, such as tests of normality and adjustment for multiple comparisons
☐	☒	A full description of the statistical parameters including central tendency (e.g. means) or other basic estimates (e.g. regression coefficient) AND variation (e.g. standard deviation) or associated estimates of uncertainty (e.g. confidence intervals)
☐	☒	For null hypothesis testing, the test statistic (e.g. *F*, *t*, *r*) with confidence intervals, effect sizes, degrees of freedom and *P* value noted<br>*Give P values as exact values whenever suitable.*
☒	☐	For Bayesian analysis, information on the choice of priors and Markov chain Monte Carlo settings
☒	☐	For hierarchical and complex designs, identification of the appropriate level for tests and full reporting of outcomes
☐	☒	Estimates of effect sizes (e.g. Cohen's *d*, Pearson's *r*), indicating how they were calculated

Our web collection on statistics for biologists contains articles on many of the points above.

Software and code

Policy information about availability of computer code

Data collection	OPTIR images and fluorescence images were collected with Photothermal Spectroscopy Corp. software PTIR Studio 4.5.
Data analysis	We used OriginPro 2022b and its embedded functions to process OPTIR spectra and performed statistical analysis. Images were color coded with ImageJ. Pearson's value was calculated with corr function in MATLAB 2021b.

For manuscripts utilizing custom algorithms or software that are central to the research but not yet described in published literature, software must be made available to editors and reviewers. We strongly encourage code deposition in a community repository (e.g. GitHub). See the Nature Portfolio guidelines for submitting code & software for further information.

Data

Policy information about availability of data

All manuscripts must include a data availability statement. This statement should provide the following information, where applicable:

- Accession codes, unique identifiers, or web links for publicly available datasets
- A description of any restrictions on data availability
- For clinical datasets or third party data, please ensure that the statement adheres to our policy

All data supporting the findings of this study are presented in this manuscript and the Supplementary Information. Source data are provided with this paper. Further information and requests for resources will be directed and will be fulfilled by the corresponding authors.

Research involving human participants, their data, or biological material

Policy information about studies with human participants or human data. See also policy information about sex, gender (identity/presentation), and sexual orientation and race, ethnicity and racism.

Reporting on sex and gender	Not applicable
Reporting on race, ethnicity, or other socially relevant groupings	Not applicable
Population characteristics	Not applicable
Recruitment	Not applicable
Ethics oversight	Not applicable

Note that full information on the approval of the study protocol must also be provided in the manuscript.

Field-specific reporting

Please select the one below that is the best fit for your research. If you are not sure, read the appropriate sections before making your selection.

- [x] Life sciences
- [ ] Behavioural & social sciences
- [ ] Ecological, evolutionary & environmental sciences

For a reference copy of the document with all sections, see nature.com/documents/nr-reporting-summary-flat.pdf

Life sciences study design

All studies must disclose on these points even when the disclosure is negative.

Sample size	No calculations were performed to predetermine sample size. Sample sizes were chosen based on prior experience, published references and the data standard deviation. The sample size of each experiment is provided in the figure captions.
Data exclusions	No data were excluded from the analysis.
Replication	All results were reproducible. Sample sizes for each experiment are indicated in the figure legends.
Randomization	Data collection was randomized to avoid operator's bias.
Blinding	Blinding is not applicable due to cell's morphological feature.

Reporting for specific materials, systems and methods

We require information from authors about some types of materials, experimental systems and methods used in many studies. Here, indicate whether each material, system or method listed is relevant to your study. If you are not sure if a list item applies to your research, read the appropriate section before selecting a response.

Materials & experimental systems

n/a	Involved in the study	
[ ]	[x]	Antibodies
[ ]	[x]	Eukaryotic cell lines
[x]	[ ]	Palaeontology and archaeology
[x]	[ ]	Animals and other organisms
[x]	[ ]	Clinical data
[x]	[ ]	Dual use research of concern
[x]	[ ]	Plants

Methods

n/a	Involved in the study	
[x]	[ ]	ChIP-seq
[x]	[ ]	Flow cytometry
[x]	[ ]	MRI-based neuroimaging

Antibodies

Antibodies used	rabbit anti-IBA1 at 1:1000 (Wako, 019-19741), rabbit anti-CD45 at 1:500 (Abcam, ab214437), donkey anti-rabbit Alexa Fluor 488 at 1:1000 (Invitrogen, A-21206), mouse anti Tuj1 at 1:500 (Sigma T8578), chicken anti GFAP at 1:1000 (abcam ab4674), Alexa Fluor 488-labeled goat anti mouse antibody at 1:500 (Thermo Fisher A11001), Alexa Fluor 647-labeled goat anti chicken antibody at 1:500 (Life tech A-21449), rabbit anti MAP2 at 1:750 (Thermo Fisher PA5-17646)
Validation	All purchased antibodies were validated for their respective application by their manufacturer.<br><br>- rabbit anti-IBA1 (Wako, 019-19741): The antibody guarantee covers the use of the antibody for Immunohistochemistry and immunocytochemistry applications. Species reactivity: Human, mouse, rat. (https://labchem-wako.fujifilm.com/us/product/detail/W01W0101-1974.html)<br><br>- rabbit anti-CD45 (Abcam, ab214437): The antibody guarantee covers the use of the antibody for flow cytometry, immunocytochemistry, immunofluorescence, immunohistochemistry, and wester blot applications. Species reactivity: Human. (https://www.abcam.com/products/primary-antibodies/cd45-antibody-ep322y-bsa-and-azide-free-ab214437.html?productWallTab=ShowAll)<br><br>- mouse anti Tuj1(Sigma T8578): The antibody guarantee covers the use of the antibody for immunocytochemistry, immunohistochemistry, immunoprecipitation, and western blot applications. Species reactivity: Human, mouse, rat. (https://www.sigmaaldrich.com/US/en/product/sigma/t8578)<br><br>- chicken anti GFAP(abcam ab4674): The antibody guarantee covers the use of the antibody for immunocytochemistry, immunohistochemistry, and western blot applications. Species reactivity: mouse, rat. (https://www.abcam.com/products/primary-antibodies/gfap-antibody-ab4674.html)<br><br>- rabbit anti MAP2(Thermo Fisher PA5-17646): The antibody guarantee covers the use of the antibody for immunocytochemistry, immunofluorescence, and western blot applications. Species reactivity: human, mouse, non-human primate, rat. (https://www.fishersci.com/shop/products/map2-polyclonal-antibody-invitrogen-4/PIPA517646)

Eukaryotic cell lines

Policy information about cell lines and Sex and Gender in Research

Cell line source(s)	Cell line H4 (male neuroglioma) was obtained from ATCC (HTB-148). CRISPRi hiPSCs (male) and iTF-microglia were provided by Martin Kampmann (UCSF). The hiPSC line F12442.4107 was kindly provided by Celeste Karch and used for organoid experiments. Sex and gender were not considered in our study.
Authentication	None of the cell lines have been authenticated.
Mycoplasma contamination	Mycoplasma contamination was routinely checked and negative results were obtained.
Commonly misidentified lines (See ICLAC register)	No commonly misidentified cell line were used in the study.
